# Mapping neglected tropical diseases: a global view

**Published:** 2013

**Authors:** Simon J Brooker, Jennifer L Smith

**Affiliations:** Professor of Epidemiology, London School of Hygiene & Tropical Medicine, London, UK.; Research Fellow, London School of Hygiene & Tropical Medicine, London, UK.

**Figure:**
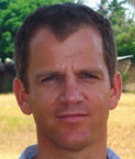
Simon J Brooker

**Figure:**
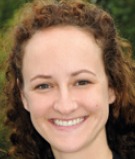
Jennifer L Smith

The word ‘mapping’ can be used to describe the collection of data that is linked to a geographical location. Today, such maps are developed using a geographical information system (GIS). This is computer software which allows the capture, storage, analysis and presentation of spatial data.

Maps are important for designing and implementing interventions targeted at the control and elimination of diseases, including neglected tropical diseases (NTDs). Forthose NTDs which are targeted for elimination, such asoncho-cerciasis and lymphatic filariasis (LF), it is essential to know where transmission occurs and when it has been successfully halted following control initiatives. For those NTDs where disease control is the goal, including trachoma, soil-transmitted helminths (STH) and schistosomiasis, interventions are most cost-effective when they are focused in areas with the highest prevalence of infection. In addition, NTD maps can be linked to population data to derive estimates of disease burden and numbers requiring intervention – information essential for estimating programme costs.

Today there are a number of GIS-based initiatives that provide information on the geographical distribution of NTDs. One of the first mapping initiatives was that of the African Programme for Onchocerciasis Control (APOC), which developed the rapid epidemiological mapping of onchocerciasis (REMO) approach. REMO quickly and cheaply identifies priority areas for community-directed treatment with ivermectin, and estimates the numbers of individuals requiring treatment. To date, 23 African countries have been mapped using this approach.

**Figure:**
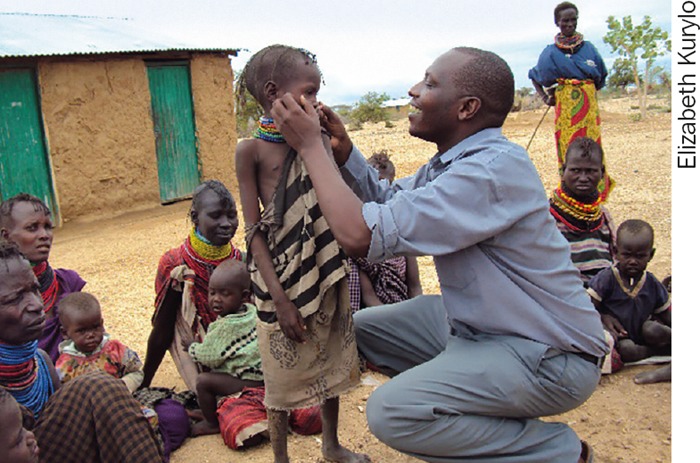
A health worker examining children during a trachoma survey. TURKANA

APOC has also mapped the distribution of *Loa loa* (tropical eye worm). These maps indicate areas where the prevalence of *Loa loa* exceeds 40%. People with high *Loa loa* parasite loads are at high risk of severe adverse events following ivermectin treatment (used for treatment of onchocerciasis and LF).

The mapping of LF has been greatly facilitated by the use of immunochroma-tographic (ICT) card tests to detect circulating *Wuchereria bancrofti* antigens. This enables large-scale assessments of the boundaries of filaria-endemic areas, so areas requiring MDA can be identified. Providing better overlay maps of onchocerciasis, *Loa loa* and LF will help the design of better integrated LF and onchocerciasis control programmes.

By contrast, the mapping of STH and schistosomiasis has been more ad hoc, with surveys conducted by a range of academic and government partners using a variety of methodologies. The *Global Atlas of Helminth Infections* (GAHI) aims to collate available data on STH and schistosomiasis, as well as LF into a single resource in order to describe their global distribution and prevalence.^1^ The assembled data are useful for a number of epidemiological purposes, such as estimating the number of people infected with these NTDs and using modern statistical methods to predict the distribution of infection in unsampled areas.^2^ The maps can also high light where further survey data are required. The GAHI web site allows users to visualise the assembled data and models, download the maps, and access the sources and underlying data.

The Global Atlas of Trachoma (GAT) is a collaborative initiative that provides an open access platform for maps of the distribution of trachoma at sub-national levels. This work is intended to provide an evidence base for allocating resources for trachoma control, including surgery and administration of azithromax, as well as to identify where further mapping is required. To address these gaps, GAT is partnered with the UK's Department for International Development (DFID)-funded Global Trachoma Mapping Project. This project uses the latest smartphone technologies to collect trachoma survey data and automatically compiles them into a linked database that allows maps to be quickly updated.

There are other NTD mapping initiatives, including the Global NTD Platform, Atlas of Human African Trypanosomiasis, Leishmaniasis e-compendium, and the WHO's global health repository, as well as efforts by WHO regional offices (see panel).

These various NTD mapping initiatives and atlases rely on contributions from the wider NTD community- revision and improvement requires further information on the prevalence of infection within countries. If you know of relevant data that could be included, or if you would like to be a partner in the initiatives, then please contact the relevant projects.

**Online resources on the geographical distribution of NTDs**

African Programme for Onchocerciasis

Control (APOC):

**www.who.int/apoc/countries/en/**

Loa loa maps:

**www.who.int/apoc/raploa/en/index.html**

Global Atlas of Helminth Infections (GAHI):

**www.thiswormyworld.org**

Global Atlas of Trachoma (GAT):

**www.trachomaatlas.org**

Global Neglected Tropical Disease platform:

**www.gntd.org**

Atlas of Human African Trypanosomiasis:

**www.who.int/trypanosomiasis_african/country/foci_AFRO/en/index.html**

Leishmaniasis e-compendium:

**apps.who.int/tools/geoserver/www/ecomp/index.html**

WHO's Global Health Repository:

**www.who.int/gho/neglected_diseases/en/index.html**

**Contact information**

Global Atlas of Helminth Infections (GAHI): **www.thiswormyworld.org/contact-us**

Global Atlas of Trachoma (GAT):

**www.trachomaatlas.org/about-us/contact-us**

SJB is supported by a Wellcome Trust Senior Fellowship in Basic Biomédical Science (098045) and acknowledges support from the Bill & Melinda Gates Foundation. JLS is supported by a grant to the International Trachoma Initiative from the Bill & Melinda Gates Foundation.
